# Long Non-Coding RNAs as Diagnostic Biomarkers for Ischemic Stroke: A Systematic Review and Meta-Analysis

**DOI:** 10.3390/genes15121620

**Published:** 2024-12-18

**Authors:** Jianwei Pan, Weijian Fan, Chenjie Gu, Yongmei Xi, Yu Wang, Peter Wang

**Affiliations:** 1 Department of Neurosurgery, The First Affiliated Hospital, Zhejiang University School of Medicine, Hangzhou 310006, China; 1508005@zju.edu.cn (W.F.); sodagucj@163.com (C.G.); 2 Institute of Genetic, Zhejiang University, Hangzhou 310007, China; xyyongm@zju.edu.cn; 3 Department of Medicine, Beijing Zhongwei Research Center, Biological and Translational Medicine, Beijing 100161, China; wangyu442@163.com

**Keywords:** epigenetic, LncRNAs, ischemic stroke, biomarker, meta-analysis

## Abstract

Ischemic stroke is a serious cerebrovascular disease, highlighting the urgent need for reliable biomarkers for early diagnosis. Recent reports suggest that long non-coding RNAs (lncRNAs) can be potential biomarkers for ischemic stroke. Therefore, our study seeks to investigate the potential diagnostic value of lncRNAs for ischemic stroke by analyzing existing research. A comprehensive literature search was conducted across the PubMed, ScienceDirect, Wiley Online Library, and Web of Science databases for articles published up to July 10, 2024. Statistical analyses were performed using Stata 17.0 software to calculate pooled sensitivity, specificity, positive likelihood ratio (PLR), diagnostic odds ratio (DOR), negative likelihood ratio (NLR), and area under the curve (AUC). Heterogeneity was explored with the Cochran-Q test and the I^2^ statistical test, and publication bias was assessed with Deeks’ funnel plot. A total of 44 articles were included, involving 4302 ischemic stroke patients and 3725 healthy controls. Results demonstrated that lncRNAs H19, GAS5, PVT1, TUG1, and MALAT1 exhibited consistent trends across multiple studies. The pooled sensitivity of lncRNAs in the diagnosis of ischemic stroke was 79% (95% CI: 73–84%), specificity was 88% (95% CI: 77–94%), PLR was 6.63 (95% CI: 3.11–14.15), NLR was 0.23 (95% CI: 0.16–0.33), DOR was 28.5 (95% CI: 9.88–82.21), and AUC was 0.88 (95% CI: 0.85–0.90). Furthermore, the results of subgroup analysis indicated that lncRNA H19 had superior diagnostic performance. LncRNAs demonstrated strong diagnostic accuracy in distinguishing ischemic stroke patients from healthy controls, underscoring their potential as reliable biomarkers. Because most of the articles included in this study originate from China, large-scale, high-quality, multi-country prospective studies are required to further validate the reliability of lncRNAs as biomarkers for ischemic stroke.

## 1. Introduction

Ischemic stroke, the most prevalent subtype of stroke, is characterized by an abrupt reduction in blood flow to a specific region of the brain, resulting in focal cerebral, spinal cord, or retinal infarction, and resulting in neurological dysfunction [1,2]. Typical symptoms of ischemic stroke include sudden headache, vertigo, unilateral weakness or paralysis, numbness, ataxia, vision disturbances, dysphagia, and speech impairments [3]. According to reports, approximately 20,000 people in China were diagnosed with ischemic stroke for the first time in 2020, with an age- and sex-standardized prevalence of 2.3%, placing a heavy burden on society [4]. Therefore, it is important to identify effective biomarkers to improve the diagnosis and treatment of ischemic stroke.

Currently, the diagnosis of ischemic stroke primarily relies on brain imaging examinations, with CT scans and MRI (magnetic resonance imaging) being the most common methods [5,6]. Although these imaging techniques have greatly improved our ability to visualize brain structures, they still have several drawbacks. The disadvantages of CT scanning include its low sensitivity and specificity for ischemic stroke, difficulty in detecting small infarcts within 6 h of onset, and patient exposure to radiation [7]. MRI scanning, on the other hand, is expensive, time-consuming, and may not be suitable for patients with metal implants, pacemakers, or claustrophobia [8]. The rapid development of high-throughput sequencing technology has facilitated the discovery of blood-based transcriptome biomarkers. Biomarkers detected using quantitative polymerase chain reaction (qPCR) technologies may aid in the diagnosis of ischemic stroke [9].

Non-coding RNAs, including long non-coding RNAs (lncRNAs), influence gene expression through interactions with chromatin and RNA-binding proteins [10,11,12]. The importance of various lncRNAs in ischemic stroke has been confirmed in numerous studies. For example, the study by Li et al. identified 44,578 abnormally expressed lncRNAs in acute ischemic stroke patients by high-throughput sequencing, with 228 upregulated and 16 downregulated lncRNAs. The upregulation of lncRNA ENSG00000226482 can promote activation of the adipokine pathway, leading to acute ischemic stroke [13]. In recent years, an increasing number of studies have highlighted the potential of lncRNA as a biomarker for disease diagnosis and prognosis. In a study using a transient middle cerebral artery occlusion/reperfusion mouse model of stroke, lncPEG11as expression was significantly raised, and silencing PEG11as inhibited autophagy, showing neuroprotective effects. This indicates that lncPEG11as could be a potential biomarker for ischemic stroke [14]. At present, lncRNAs have been incorporated into clinical practice as biomarkers. For example, the lncRNA prostate cancer antigen 3 (PCA3) has been approved by the U.S. Food and Drug Administration as a diagnostic biomarker for prostate cancer and is detectable in urine extracellular vesicles [15]. Several lncRNAs are either undergoing clinical trials or have been patented. Recently, a phase 3 clinical trial identified an integrated mRNA-lncRNA signature with the potential to personalize adjuvant chemotherapy for patients with operable triple-negative breast cancer [16]. Additionally, an ongoing clinical trial is exploring the role of lncRNA CCAT1 as a diagnostic marker for colorectal cancer [17].

Although several studies have highlighted the significant contribution of dysregulated lncRNAs in the diagnosis of ischemic stroke, these studies vary in research techniques, statistical analysis methods, and cut-off values. Therefore, we conducted a comprehensive systematic review and meta-analysis of existing research to evaluate the overall diagnostic accuracy in ischemic stroke patients.

## 2. Results

### 2.1. Search Results and Study Selection

**Figure 1:**
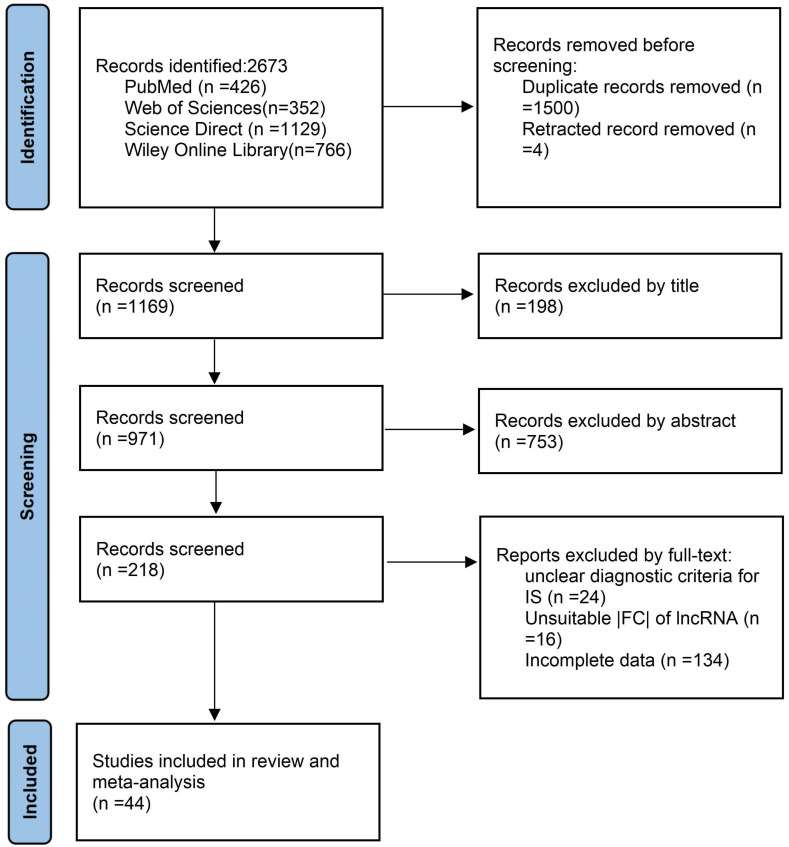
Flow diagram of study search and selection.

The initial search yielded 2673 results. After removing duplicates, 1169 articles remained and were screened based on their titles and abstracts. A total of 951 articles were removed as irrelevant, leaving 218 articles suitable for full-text analysis. Ultimately, 44 studies met the inclusion criteria and were considered for a systematic review and meta-analysis (Figure 1).

### 2.2. Characteristics of Included Studies

This study included lncRNA data from 44 studies, encompassing 4302 ischemic stroke patients and 3725 healthy controls. All included studies utilized qRT-PCR to evaluate lncRNA expression. In terms of lncRNA expression profiles, 34 studies investigated individual lncRNAs, while 10 studies examined combined lncRNAs. Among the 40 lncRNAs, 26 were reported to be upregulated, including lncRNA H19, lncRNA growth arrest-specific transcript 5 (GAS5), lncRNA plasmacytoma variant translocation 1 (PVT1), and lncRNA taurine-upregulated gene 1(TUG1), which were reported in more than one study. Twelve lncRNAs were downregulated in ischemic stroke patients, with lncRNA metastasis-associated lung adenocarcinoma transcript-1 (MALAT1) being reported in more than one study. There was controversy over the expression levels of two lncRNAs: lncRNA antisense non-coding RNA in the INK4 locus (ANRIL) and lncRNA myocardial infarction-related transcript (MIAT) (Table 1). Table 2 summarizes the detailed information of the reported lncRNAs, including their names, fold change (FC), *p*-values, area under the receiver operating characteristic curve (AUC), cutoff values, sensitivity, and specificity. Among these, 17 studies reported the potential of lncRNAs as biomarkers of ischemic stroke and were included in the subsequent meta-analyses.

### 2.3. Quality Assessment of Studies Included in Meta-Analysis

**Figure 2:**
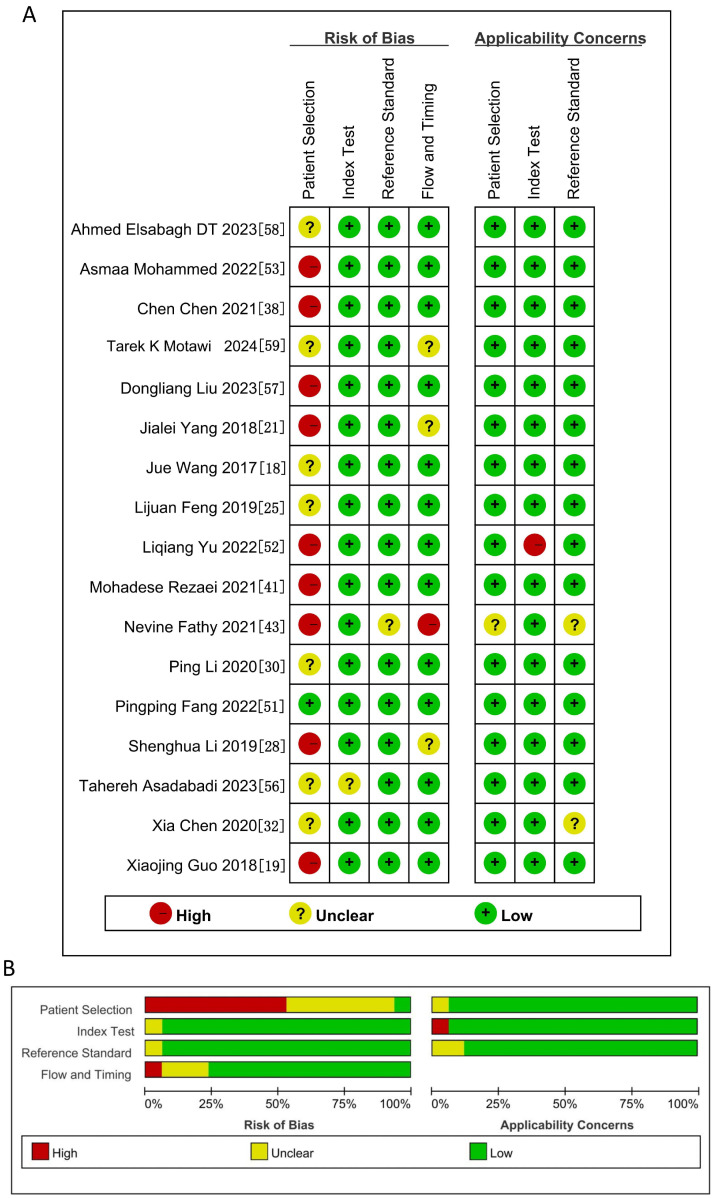
Risk of bias assessment of eligible studies using QUADAS-2. (**A**) Summary of bias risk items in the QUADAS-2 quality assessment. (**B**) Percentile of risk of bias in the QUADAS-2 quality assessment [18,19,21,25,28,30,32,38,41,43,51,52,53,56,57,58,59].

The quality of the literature was assessed by the quality assessment of diagnostic accuracy studies 2 (QUADAS-2) tool to meet the inclusion criteria. We determined that the patient selection domain had a higher risk of bias, as all studies were designed as controlled trials. It is unclear whether inappropriate exclusions were avoided. For the index test, reference standard, and flow and timing domains, we assessed the risk of bias to be low (Figure 2A,B).

### 2.4. Diagnostic Accuracy of lncRNA for IS

**Figure 3:**
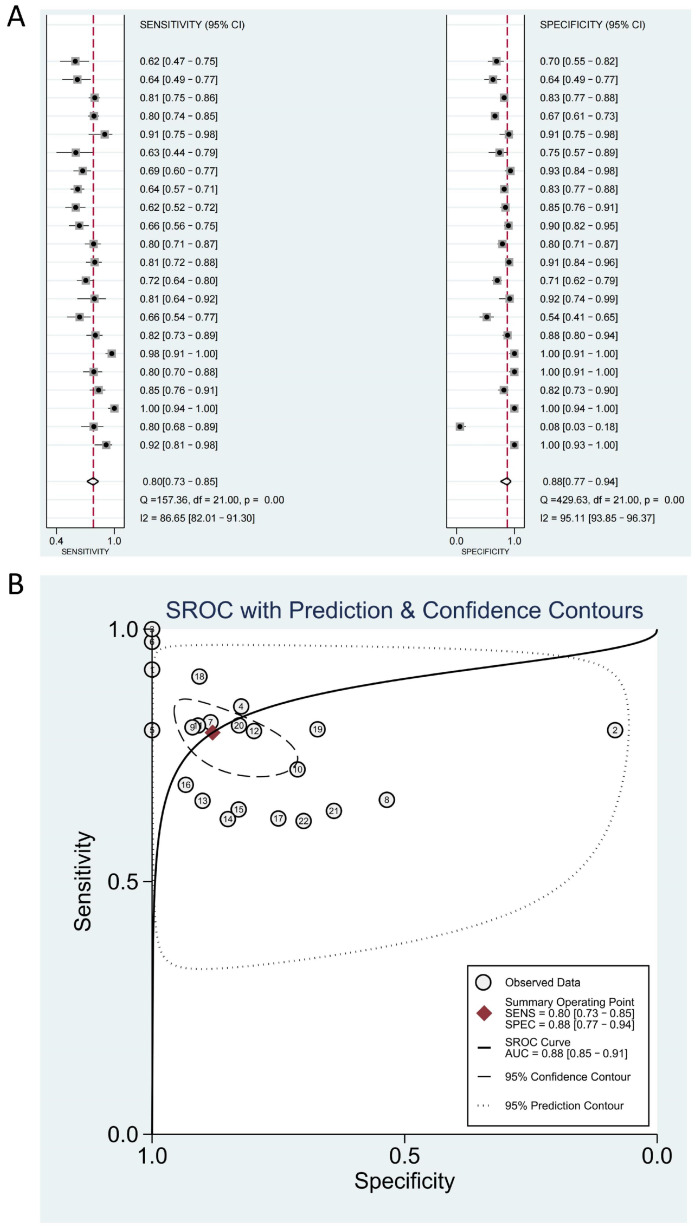
(**A**) Forest plot showing the pooled sensitivity and specificity of lncRNAs in diagnosing ischemic stroke. Squares represent individual studies, while line segments indicate the 95% confidence interval (CI) for each study. The center of the diamond and the red dashed line represent the pooled effect size, and the width of the diamond corresponds to the 95% CI of the pooled results. (**B**) Summary receiver operating characteristic (SROC) curve with the 95% confidence and prediction contours. The *Y*-axis represents sensitivity, and the *X*-axis represents specificity. Numbers represent individual studies, and the curves depict combined diagnostic performance.

The summary analysis of sensitivity and specificity of lncRNAs was performed. The combined sensitivity of lncRNAs for diagnosing ischemic stroke was 79% (95% CI: 73–84%; Cochran’s Q: 155.50; I^2^ index:85.85%). The combined specificity was 88% (95% CI: 77–94%; Cochran’s Q: 426.26; I^2^ index: 94.84%) (Figure 3A). ROC analysis indicated that lncRNAs have high accuracy as diagnostic factors for ischemic stroke (AUC = 0.88; 95% CI: 0.85–0.90) (Figure 3B). The combined positive likelihood ratio (PLR) estimate was 6.63 (95% CI: 3.11–14.15; Cochran’s Q: 728.29; I^2^ index = 96.49%), and the combined negative likelihood ratio (NLR) estimate was 0.23 (95% CI: 0.16–0.33; Cochran’s Q: 207.85; I^2^ index = 89.90%). The combined diagnostic score estimate was 3.35 (95% CI: 2.29–4.41; Cochran’s Q: 232.64; I^2^ index = 90.97%, Figure S1A), and the combined diagnostic odds ratio (DOR) estimate was 28.50 (95% CI: 9.88–82.21; Cochran’s Q: 1.4e+22; I^2^ index = 100%, Figure S1B).

### 2.5. Clinical Applicability of lncRNA for Diagnosing IS

**Figure 4:**
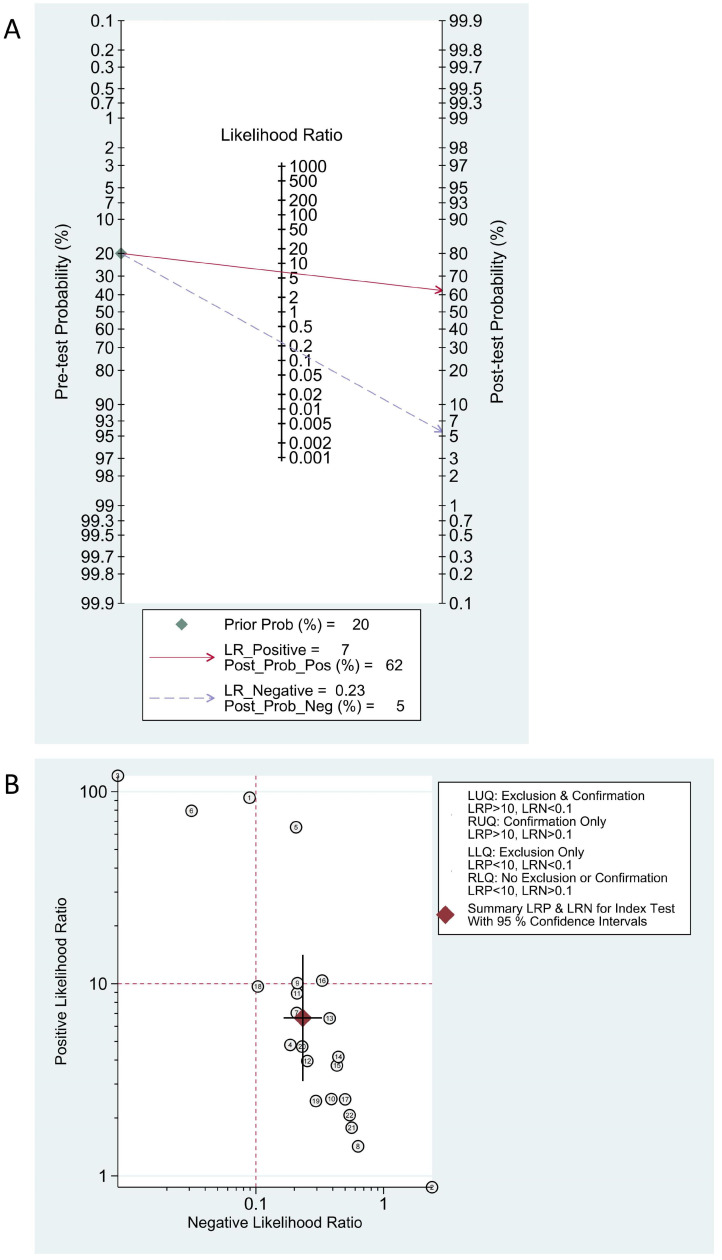
Fagan nomogram (**A**) and likelihood ratio scattergram (**B**) are illustrated. (**A**) If two values are known, the nomogram can be used to calculate a third value. (**B**) The ordinate represents the positive likelihood ratio, indicating the likelihood of a positive result in a patient compared to a non-patient. The abscissa represents the negative likelihood ratio, indicating the likelihood of a negative result in a patient compared to a non-patient.

Fagan’s nomogram and the likelihood ratio scatter plot were used to assess the clinical utility of lncRNAs in the diagnosis of ischemic stroke. The Fagan’s nomogram results showed that, with a pre-test probability set to 20%, the PLR and NLR values of lncRNA were 0.62 and 0.05, respectively (Figure 4A). The likelihood ratio scatter plot revealed that the studies by Ahmed Elsabagh DT et al. [58] (lncRNA GAS5), Asmaa Mohammed et al. [53] (lncRNA NBAT1), and Tarek K Motawi et al. [59] (lncRNA H19) were in the upper left quadrant, indicating that these two lncRNAs can be used to both exclude and confirm ischemic stroke (Figure 4B).

### 2.6. Subgroup Analysis and Meta-Regression

**Figure 5:**
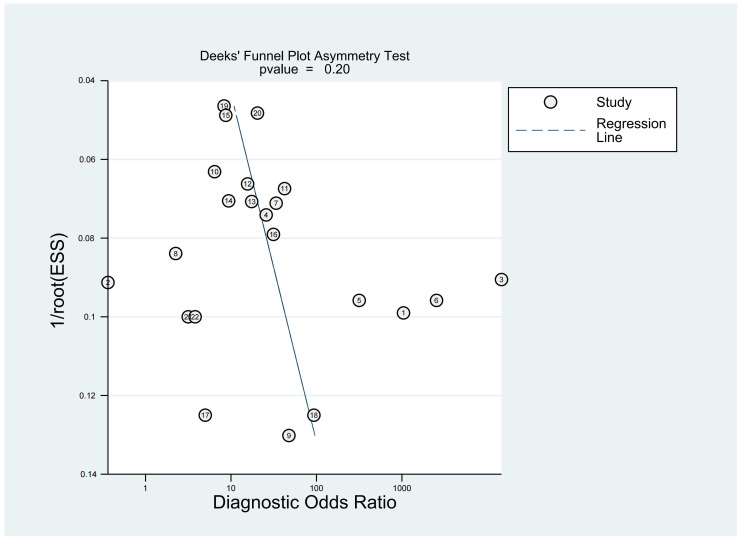
Deeks’ funnel plot for publication bias analysis. A *p*-value > 0.05 indicates no significant publication bias.

Sensitivity analysis demonstrated that individual studies often exerted a disproportionate impact on overall heterogeneity (Figure S2). Due to significant heterogeneity (I^2^ > 50%, *p* < 0.05) across all diagnostic performance parameters, including sensitivity, specificity, PLR, and NLR, meta-regression and subgroup analyses were conducted (Table 3). These analyses aimed to identify the sources of heterogeneity among studies and examine various research characteristics, including sample size, sampling time, type of lncRNA, cutoff value establishment, and biological species. In the subgroup analysis, studies sampled within 48 h showed higher diagnostic sensitivity of 0.81 (95%CI: 0.73–0.89). Compared to other lncRNAs, lncRNA H19 exhibited higher diagnostic sensitivity (0.89, 95% CI: 0.80–0.98) and specificity (0.96, 95% CI: 0.87–1.00), indicating that lncRNA H19 had relatively high diagnostic performance for ischemic stroke. The meta-regression analysis of lncRNA markers revealed that sample size and biological species were sources of heterogeneity for sensitivity between studies. In addition, the year of publication, type of lncRNA, and cutoff value establishment did not affect the sensitivity and specificity of lncRNA detection for ischemic stroke (Figure S3). Deeks’ funnel plot asymmetry test was conducted to assess potential publication bias. A *p*-value of 0.20 suggests that the included studies showed no significant signs of publication bias (Figure 5).

## 3. Discussion

**Figure 6:**
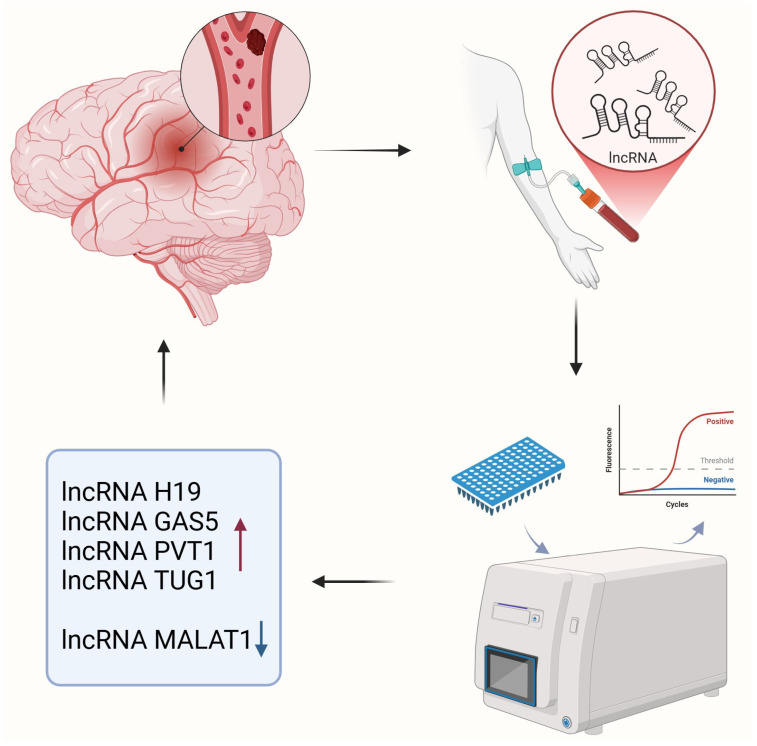
LncRNAs as diagnostic markers for ischemic stroke. Created using https://BioRender.com (accessed on 3 December 2024).

As populations age, the burden of ischemic stroke is increasing globally [6,60,61]. Therefore, it is critical to develop accurate and timely biomarkers to improve the early prediction and diagnosis of ischemic stroke. LncRNAs are highly stable in body fluids, allow for easy non-invasive detection, and can act as diagnostic biomarkers for ischemic stroke [62,63]. This study screens a total of 44 studies (17 of which were used for meta-analysis) involving 4302 patients with ischemic stroke and 3725 healthy controls. Multiple studies reported differential expression of lncRNAs H19, GAS5, PVT1, TUG1, and MALAT1 in ischemic stroke. The included studies were of relatively high quality and showed no significant publication bias. In this study, the combined sensitivity of lncRNAs in the diagnosis of ischemic stroke was 79%, the combined specificity was 88%, and the AUC was 0.88. These results suggest that lncRNAs have moderate levels of comprehensive sensitivity and specificity as diagnostic biomarkers for ischemic stroke (Figure 6).

Our study showed that the combined positive LR was 6.63, indicating that the likelihood of detecting lncRNA dysregulation in ischemic stroke patients was 6.63 times higher than that in the healthy control group. Meanwhile, the merged negative LR was 0.23, indicating that individuals with normal lncRNA expression levels have a 23% chance of being misclassified as having ischemic stroke. The DOR reflected the degree of correlation between the diagnostic test results and diseases, with a larger value indicating a better discriminative effect. In this study, the DOR was 28.5, underscoring the capability of lncRNAs to effectively differentiate between ischemic stroke patients and healthy participants.

According to the subgroup analysis, lncRNA H19 exhibited higher diagnostic sensitivity and specificity compared to other types of lncRNA. LncRNA H19 is located on human chromosome 11, and its polymorphism has been linked to susceptibility to small vessel ischemic stroke in the Han population of northern China. This marked it a promising biomarker for ischemic stroke [64]. In the pathogenesis of ischemic stroke, lncRNA H19 regulates ACP5 expression by interaction with ACP5. It affects the proliferation and regulates apoptosis of arterial endothelial cells, thereby inducing atherosclerosis and leading to ischemic stroke [23]. LncRNA H19 also has potential as a therapeutic target. Studies have reported that circulating levels of lncRNA H19 are increased in ischemic stroke patients, and targeted inhibition of H19 can alleviate neurological deficits and reduce cerebral infarction volume in MCAO/R rats [27]. Additionally, high expression of lncRNA GAS5 in ischemic stroke patients was observed. Located on chromosome 1q25.1, the promoter region rs145204276 insertion-deletion variant of lncRNA GAS5 increases the risk of ischemic stroke in the Han population [35].

Subgroup analyses identified study sample size, sampling time, and sample types as key contributors to the observed high heterogeneity. A sufficient sample size improves the generalizability of conclusions and enhances representativeness while reducing errors caused by subjective bias. Consequently, the results of the subgroup analysis based on sample size were consistent with the preselected findings, showing significantly better diagnostic sensitivity in studies with more than 100 cases. Sampling time after stroke was another critical factor affecting the accuracy of lncRNA-based diagnosis. Variability in sample collection times among the included studies also contributed to heterogeneity. For example, it has been reported that the expression level of lncRNA XIST in patients with ischemic stroke significantly decreased 48 h after onset and significantly increased 7 days after onset compared with healthy controls [36]. This indicates that lncRNA expression levels may vary over time, similar to changes in thrombin levels following the onset of myocardial infarction. To enable the clinical application of lncRNAs in stroke diagnosis, it is necessary to develop diagnostic criteria tailored to the time of onset, including the use of different lncRNAs or varying expression levels. Most of the included studies used serum or plasma as the test sample, with only one study using PBMCs. Our subgroup analysis categorized studies based on the type of biological sample. The findings revealed that biological sample types were a major source of heterogeneity, with studies using plasma showing significantly lower diagnostic sensitivity. This highlights the importance of ensuring the reliability and stability of both test samples and methods in future research.

In addition, there may have been biological and methodological heterogeneity among studies. Biologically, there are variations in patient demographics, including gender and age, across studies. For example, the proportion of male participants in the study by Asmaa Mohammed et al. was only 47.5% [53], while it was high as 78.0% in the study of by Yi Zhang et al. [33]. Similarly, the age of patients varied, with some studies including younger participants (mean age: 56.27 ± 11.00 years) and others including older participants (mean age: 67.7 ± 10.0 years). Age-related physiological changes may influence the degree of lncRNA expression, potentially contributing to variability in results. In terms of methodology, among the studies included in the meta-analysis, only the study by Pingping Fang et al. was prospective [51], while the rest were retrospective. Differences in study design may have influenced the results. Furthermore, variation in the use of blinding measures were observed. Blinded studies are more prone to selection bias and evaluation bias, which may have affected the consistency of findings.

The clinical application of lncRNAs as diagnostic markers remains underexplored. Factors such as population diversity, underlying pathological conditions, and stroke subtypes may affect lncRNA expression. For instance, previous studies have shown that H19 can serve as a biomarker not only for ischemic stroke, but also for gastric, lung, and bladder cancers and other diseases [65]. To establish the disease specificity of lncRNAs as diagnostic markers for ischemic stroke, extensive research is required. First, an in-depth investigation of the intrinsic molecular mechanism linking lncRNAs to ischemic stroke is necessary to clarify the source of specificity. Second, clinical trials should evaluate the stability of lncRNAs in various clinical settings, accounting for factors such as drug use and complications. Finally, the study population should be stratified by age, sex, ethnicity, time of onset, and other relevant factors to ensure the specificity of lncRNAs within distinct subgroups. In addition, attention must be given to technical specifications, as well as ethical and legal requirements, during the process of clinical translation. All studies included in the meta-analysis utilized qRT-PCR to measure lncRNA expression levels. qRT-PCR technology offers several advantages, including simplicity of operation, ease of standardization, rapid detection, and broad applicability. These features make qRT-PCR a promising tool for facilitating the clinical translation of lncRNAs as diagnostic biomarkers for ischemic stroke.

This study also had some other limitations. Firstly, differences in the critical values of biomarkers and the lack of reported critical values in some studies led to relatively limited data extraction, which may contribute to potential heterogeneity. Secondly, there was controversy over the expression levels of lncRNA ANRIL and lncRNA MIAT in ischemic stroke patients across different studies, necessitating more clinical and cellular experiments to clarify their roles. Moreover, most of the included studies were conducted in China, which limits the generalizability of our findings to a global population. To mitigate these limitations, future research should focus on conducting larger-scale, multicenter prospective studies with standardized cutoff values, increased global representation, and reduced potential bias. Such efforts are essential to further validate the clinical utility of lncRNAs as diagnostic biomarkers for ischemic stroke and enhance their applicability across diverse populations. In summary, this study identified a set of lncRNAs as biomarkers for ischemic stroke, demonstrating good diagnostic efficacy in distinguishing ischemic stroke patients from healthy controls and showing potential as early diagnostic tools for ischemic stroke. Among them, lncRNA H19 showed high potential in the diagnosis of ischemic stroke. However, future large-scale and high-quality prospective studies need to be carefully designed, with standardized cutoff values, to promote the transformation of these biomarkers into clinically significant diagnostic tools for ischemic stroke.

## 4. Methods

This study was conducted in accordance with the Preferred Reporting Items for Systematic Reviews and Meta Analyses (PRISMA) guideline and registration protocol. This systematic review protocol is registered on the PROSPERO database (registration No. CRD42024568376).

### 4.1. Search Strategy

Before 10 July 2024, a systematic search was conducted using the keywords [“ischemic stroke”] and [“Long non coding RNA” or “lncRNA”] in the PubMed, ScienceDirect, Wiley Online Library, and Web of Science databases, without language limitations. PubMed is a biomedical information retrieval system provided by the U.S. National Library of Medicine. It collects medical literature from around the world and serves as an important resource for meta-analysis. ScienceDirect database hosts a vast collection of high-quality academic journals and is a key source for scholars seeking academic materials. Wiley Online Library is one of the largest and most comprehensive online databases, and offers extensive full-text search capabilities across various disciplines. Web of Science is a multidisciplinary academic database and provides access to literature spanning multiple fields, including medicine and biological sciences. These databases were selected for their breadth and reliability to enhance the accuracy and comprehensiveness of our search. In addition, comprehensive reviews and meta-analyses were excluded. After selecting the final article, a manual search was performed by reviewing the references of relevant articles to avoid omissions.

### 4.2. Study Selection

After removing duplicate results, two researchers independently reviewed the titles and abstracts of the remaining articles based on the following inclusion and exclusion criteria. Disputes were resolved through discussion with a third party.

Inclusion criteria: ① Patients with ischemic stroke included in the study were diagnosed with ischemic stroke for the first time, and diagnosis met international standards, including confirmation through head magnetic resonance imaging or computed tomography. ② The study detected lncRNA expression in ischemic stroke patients and healthy controls using real-time quantitative reverse transcription polymerase chain reaction (qRT-PCR), RNA microarray, or RNA sequencing techniques. ③ The reported lncRNA with | fold changes (FC) | > 1.5 or <0.6, with *p* < 0.05.

Exclusion criteria: ① Review and meta-analysis articles; ② studies that do not provide data on the number of ischemic stroke individuals and healthy control individuals; ③ studies that do not report or provide insufficient data to calculate the FC of differentially expressed lncRNAs; ④ participants in the study who had other diseases, excluding hypertension and diabetes.

### 4.3. Data Extraction and Outcome Definition

The extracted data from the selected study included the first author’s name, publication year, sample size, lncRNA and its expression profile (FC, *p*-value, upregulation/downregulation), detection sample, and detection method. Two authors independently performed the data extraction process, which was then double-checked by a third researcher to ensure accuracy. When FC values were not provided in the article or Supplementary Materials, WebPlotDigitizer (version 4.5) was used to extract statistical data from the chart [66].

### 4.4. Quality Assessment

The QUADAS-2 tool was used to evaluate the risk of bias in the included studies. This tool assesses four main areas: patient selection, indicator testing, reference standards, and process and timing. Each area is rated as “low”, “high”, or “unclear” based on the risk of bias. The evaluation was performed by two qualified team members, and the final scores were consistent. This process was carried out using the Review Manager (RevMan5.4) software.

### 4.5. Statistical Analysis

All statistical analyses were performed using STATA (17.0). We first extracted specificity and sensitivity values from each study, then calculated true positive (TP), true negative (TN), false positive (FP), and false negative (FN) values based on the sample size of ischemic stroke patients and control groups. A bivariate random effects model and a summary receiver operating characteristic (SROC) curve were applied for the analysis. The Midas module was employed to calculate the summary diagnostic performance of lncRNAs, including sensitivity, specificity, PLR, NLR, DOR, and area under the receiver operating characteristic curve (AUC). Statistical heterogeneity was assessed using Cochrane’s Q-test and Higgins’ I-squared test (with I^2^ > 50% indicating heterogeneity). Publication bias was investigated through Deeks’ funnel plot asymmetry test, with a *p*-value < 0.05 indicating the presence of publication bias. All final outcomes were presented with a 95% confidential interval. We conducted subgroup analyses considering various study characteristics, including sample size, year of publication, type of lncRNA, cutoff value establishment, and biological species. The robustness of the results was evaluated by a leave-one-out sensitivity analysis.

## Figures and Tables

**Table 1:** Baseline characteristics of included studies.

Study, Year	Detection Method	Detected Sample	Case (Con, N)	Case (IS, N)	Up lncRNA	Down lncRNA
Jue Wang, 2017 [18]	qRT-PCR	plasma/neutrophil/lymphocyte	25	36	1	/
Xiaojing Guo, 2018 [19]	Microarray	peripheral blood	10	10	560	690
	qRT-PCR		50	50	1	1
Qi-Wen Deng, 2018 [20]	Microarray	PBMC	179	206	70	128
	qRT-PCR		32	32	3	/
Jialei Yang, 2018 [21]	qRT-PCR	peripheral blood	71	71	1	/
Kui Zhang, 2019 [22]	qRT-PCR	Serum	107	126	1	/
Yujing Huang, 2019 [23]	qRT-PCR	Serum	85	85	1	/
Jue Wang, 2019 [24]	qRT-PCR	plasma	25	40	1	/
Lijuan Feng, 2019 [25]	qRT-PCR	plasma	125	126	/	1
Shijia Yu, 2019 [26]	qRT-PCR	plasma	40	42	1	/
Zhipeng Xiao, 2019 [27]	qRT-PCR	plasma	25	40	1	/
Shenghua Li, 2019 [28]	RNA-seq	peripheral blood	3	3	428	791
	qRT-PCR		32	32	/	2
Yiming Deng, 2020 [29]	qRT-PCR	Serum	30	55	1	/
Jia Li, 2020 [13]	RNA-seq	peripheral blood	20	20	228	16
	qRT-PCR		30	30	5	1
Ping Li, 2020 [30]	qRT-PCR	plasma	210	210	1	/
Jingjing Lu, 2020 [31]	qRT-PCR	plasma	30	30	1	/
Xia Chen, 2020 [32]	qRT-PCR	plasma	215	215	1	/
Yi Zhang, 2020 [33]	qRT-PCR	plasma	320	320	1	/
Hongbo Ren, 2020 [34]	qRT-PCR	plasma	120	120	/	1
Fu Deng, 2021 [35]	qRT-PCR	PBMC	95	98	1	/
Cong Wang, 2021 [36]	qRT-PCR	peripheral blood	60	77	/	1
Bin Ren, 2021 [37]	qRT-PCR	blood CD4 T cells	160	160	1	/
Chen Chen, 2021 [38]	qRT-PCR	plasma	85	98	1	/
Liyan Liu, 2021 [39]	qRT-PCR	plasma	30	30	1	/
Yaxuan Sun, 2021 [40]	qRT-PCR	peripheral blood	10	10	1	/
Mohadese Rezaei, 2021 [41]	qRT-PCR	peripheral blood	114	114	1	/
Yuqin Chen, 2021 [42]	qRT-PCR	plasma	15	15	/	1
Nevine Fathy, 2021 [43]	qRT-PCR	Serum	100	100	1	1
Chunping Liu, 2021 [44]	qRT-PCR	PBMC	100	170	1	/
Wen Jiang, 2022 [45]	Microarray	Serum	20	10	1318	2023
	qRT-PCR		5	5	/	1
Jiao Huang, 2022 [46]	qRT-PCR	peripheral blood	153	159	1	/
Nianping Feng, 2022 [47]	qRT-PCR	plasma	68	43	/	1
Chen Xie, 2022 [48]	qRT-PCR	PBMC	60	120	/	1
Pu Xiang, 2022 [49]	qRT-PCR	peripheral blood	46	46	1	/
Gang Wang, 2022 [50]	qRT-PCR	PBMC	120	241	/	1
Pingping Fang, 2022 [51]	qRT-PCR	plasma	60	120	1	/
Liqiang Yu, 2022 [52]	qRT-PCR	PBMC	110	110	1	/
Asmaa Mohammed, 2022 [53]	qRT-PCR	Serum	60	60	2	/
Gang Wang, 2022 [54]	qRT-PCR	PBMC	50	102	1	/
Jianquan You, 2022 [55]	qRT-PCR	plasma	30	30	/	1
Tahereh Asadabadi, 2023 [56]	qRT-PCR	peripheral blood	232	232	1	/
Dongliang Liu, 2023 [57]	qRT-PCR	plasma	95	103	1	/
Ahmed Elsabagh DT, 2023 [58]	qRT-PCR	Serum	50	50	1	/
Tarek K Motawi, 2024 [59]	qRT-PCR	Serum	40	80	1	1

**Table 2:** Detailed features of lncRNAs in ischemic stroke.

Study, Year	lncRNA	Regulation	FC	*p*	AUC	Cut-Off	Sensitivity	Specificity
Jue Wang, 2017 [18]	lncRNA H19	Up	3.500 a (plasma)	*p* < 0.001	0.910	NA	80.6	92.0
			2.00 a (neutrophil)	*p* < 0.01	0.776	NA	55.6	92.0
			3.636 a (lymphocyte)	*p* < 0.01	0.787	NA	75.0	72.0
Jialei Yang, 2018 [21]	lncRNA ANRIL	Up	2.306 a	*p* = 0.002	0.642	NA	66.3	53.8
Qi-Wen Deng, 2018 [20]	lncRNA-DHFRL1-4	Up	3.17 b	*p* < 0.01	0.711	12.77	68.7	71.9
Xiaojing Guo, 2018 [19]	lncRNA-ENST00000568297	Up	2.965 b	*p* < 0.05	0.733	0.001	64.8	63.6
	lncRNA-NR_046084	Up	1.902 b	*p* < 0.01	0.690	0.002	61.5	69.2
Yujing Huang, 2019 [23]	lncRNA H19	Up	2.245 a	*p* < 0.001	NA	NA	NA	NA
Jue Wang, 2019 [24]	lncRNA H19	Up	3.452 a	*p* < 0.001	NA	NA	NA	NA
Lijuan Feng, 2019 [25]	lncRNA ANRIL	Down	0.360 b	*p* < 0.001	0.759	NA	72.2	71.2
Zhipeng Xiao, 2019 [27]	lncRNA H19	Up	3.899 a	*p* < 0.05	NA	NA	NA	NA
Kui Zhang, 2019 [22]	lncRNA ANRIL	Up	2.813 b	*p* < 0.001	0.829	NA	NA	NA
Shijia Yu, 2019 [26]	lncRNA KCNQ1OT1	Up	2.430 a	*p* < 0.001	NA	NA	NA	NA
Shenghua Li, 2019 [28]	lncRNA-C14orf64	Down	0.543 a	*p* < 0.05	0.74	NA	63	75
	lncRNA-AC136007.2	Down	0.283 a	*p* < 0.01	0.94	NA	90.63	90.63
Jingjing Lu, 2020 [31]	lncRNA PVT1	Up	2.099 a	*p* < 0.001	NA	NA	NA	NA
								
Ping Li, 2020 [30]	lncRNA NEAT1	Up	2.135 b	*p* < 0.001	0.804	1.471	64.3	82.9
Yi Zhang, 2020 [33]	lncRNA-ITSN1-2	Up	2.128 a	*p* < 0.001	0.804	NA	NA	NA
Hongbo Ren, 2020 [34]	lncRNA-MALAT1	Down	0.398 a	*p* < 0.001	0.791	NA	NA	NA
Jia Li, 2020 [13]	SNHG15	Up	3.02 b	*p* < 0.001	0.756	9.39	59.4	43.8
	lncRNA-FAM98A-3	Up	2.19 b	*p* < 0.05	0.659	7.37	59.4	28.2
	ENSG00000269900	Up	7.339 a	*p* < 0.01	NA	NA	NA	NA
	ENSG00000196559	Up	5.629 a	*p* < 0.01	NA	NA	NA	NA
	ENSG00000202198	Up	8.711 a	*p* < 0.01	NA	NA	NA	NA
	ENSG00000226482	Up	8.881 a	*p* < 0.01	NA	NA	NA	NA
	ENSG00000260539	Up	4.256 a	*p* < 0.01	NA	NA	NA	NA
	XLOC_013994_2	Down	0.376 a	*p* < 0.01	NA	NA	NA	NA
Xia Chen, 2020 [32]	lncRNA HULC	Up	2.421 a	*p* < 0.001	0.876	1.508	80.9	82.8
Chen Chen, 2021 [38]	lncRNA CASC15	Up	1.523 a	*p* < 0.001	0.902	1.339	84.7	82.4
Liyan Liu, 2021 [39]	lncRNA AK139328	Up	3.480 a	*p* < 0.001	NA	NA	NA	NA
Bin Ren, 2021 [37]	lncRNA UCA1	Up	2.955 b	*p* < 0.001	NA	NA	NA	NA
Fu Deng, 2021 [35]	lncRNA GAS5	Up	3.185 a	*p* < 0.01	NA	NA	NA	NA
Yaxuan Sun, 2021 [40]	SNHG5	Up	1.886 a	*p* < 0.01	NA	NA	NA	NA
Cong Wang, 2021 [36]	lncRNA XIST	Down	0.560 a	*p* < 0.001	NA	NA	NA	NA
Mohadese Rezaei, 2021 [41]	lncRNA H19	Up	3.804 a	*p* < 0.001	0.874	1.246	79.49	79.49
Yuqin Chen, 2021 [42]	lncRNA OIP5-AS1	Down	0.406 a	*p* < 0.001	NA	NA	NA	NA
Nevine Fathy, 2021 [43]	lncRNA MALAT1	Down	4.54 b	*p* < 0.001	0.8	<0.75	66	90
	lncRNA ANRIL	Up	2.25 b	*p* < 0.01	0.74	>1.003	62.5	85
Chunping Liu, 2021 [44]	lncRNA-MEG3	Up	2.216 a	*p* < 0.001	0.874	NA	NA	NA
Chen Xie, 2022 [36]	lncRNA SNHG16	Down	0.501 b	*p* < 0.001	NA	NA	NA	NA
Wen Jiang, 2022 [45]	lncRNA ENST00000530525	Down	0.069 a	*p* < 0.05	NA	NA	NA	NA
Gang Wang, 2022 [50]	lncRNA-ZFAS1	Down	0.417 a	*p* < 0.001	0.845	NA	NA	NA
Pingping Fang, 2022 [51]	lncRNA-GAS5	Up	2.492 a	*p* < 0.001	0.893	1.865	69.2	93.3
Liqiang Yu, 2022 [52]	lncRNA-PVT1	Up	2.954 a	*p* < 0.001	0.916	1.922	80.9	90.9
Asmaa Mohammed, 2022 [53]	TUG1	Up	1.503 b	*p* < 0.001	0.733	0.853	80	8.3
	NBAT1	Up	13.259 b	*p* < 0.001	1.00	1.45	100	100
Gang Wang, 2022 [54]	lncRNA-ITSN1-2	Up	2.581 b	*p* < 0.001	0.927	NA	NA	NA
Nianping Feng, 2022 [47]	ADAMTS9-AS2	Down	0.556 a	*p* < 0.001	NA	NA	NA	NA
Jianquan You, 2022 [55]	lncRNA WT1-AS	Down	0.374 a	*p* < 0.01	NA	NA	NA	NA
Jiao Huang, 2022 [46]	lncRNA SERPINB9P1	Down	0.564 a	*p* < 0.05	NA	NA	NA	NA
Pu Xiang, 2022 [49]	lncRNA TUG1	Up	1.863 a	*p* < 0.001	NA	NA	NA	NA
Dongliang Liu, 2023 [57]	lncRNA NORAD	Up	1.539 a	*p* < 0.001	0.903	NA	81.60	88.40
Ahmed Elsabagh DT, 2023 [58]	lncRNA GAS5	Up	11.31 b	*p* < 0.001	0.920	2.11	92.0	100.0
Tahereh Asadabadi, 2023 [56]	lncRNA MIAT	Up	3.018 a	*p* < 0.001	0.82	NA	80.17	67.24
Tarek K Motawi, 2024 [59]	lncRNA MIAT	Down	0.569 b	*p* < 0.001	0.819	1.21	80	100
	lncRNA H19	Up	23.19 b	*p* < 0.001	0.975	0.24	97.5	100

Notes. FC, fold change; *p, p* value; AUC, area under curve, NA, not reported. For FC, “a” represents the data provided by the article, and “b” represents the data retrieved from the graph using WebPlotDigitizer.

**Table 3:** Subgroup analysis and meta-regression.

Subgroups	No. of Studies	Sensitivity [95% CI]	*p* Value	Specificity [95% CI]	*p* Value
Sample size					
≤100	6	0.78 [0.66–0.90]	0.03	0.89 [0.74–1.00]	0.96
>100	16	0.80 [0.74–0.86]		0.88 [0.78–0.98]	
Type of lncRNA					
lncRNA H19	3	0.89 [0.80–0.98]	0.68	0.96 [0.87–1.00]	0.10
Other types	19	0.78 [0.72–0.84]		0.86 [0.76–0.96]	
cut-off value establishment					
Reported	15	0.80 [0.74–0.87]	0.06	0.91 [0.83–0.99]	0.51
Not reported	7	0.78 [0.67–0.88]		0.80 [0.59–1.00]	
Sampling time					
Within 48 h	10	0.81 [0.73–0.89]	0.03	0.86 [0.73–1.00]	0.46
More than 48 h or not reported	12	0.78 [0.70–0.86]		0.90 [0.79–1.00]	
biological specimen					
Plasma	6	0.76 [0.64–0.88]	0.01	0.86 [0.68–1.00]	0.59
Others	16	0.81 [0.74–0.87]		0.89 [0.79–0.98]	

## Data Availability

Data are contained within the article or Supplementary Materials.

## Supplementary Materials

The following supporting information can be downloaded at: https://www.mdpi.com/article/10.3390/genes15121620/s1, Figure S1: Diagnostic Accuracy of lncRNA for IS. Figure S2: Sensitivity analysis. Figure S3: Meta-regression analysis for sensitivity and specificity.

## Abbreviation

ANRIL	antisense non-coding RNA in the INK4 locus;
AUC	area under the receiver operating characteristic curve;
DOR	diagnostic odds ratio;
FC	fold change;
FN	false negative;
FP	false positive;
GAS5	growth arrest-specific transcript 5;
MALAT1	metastasis-associated lung adenocarcinoma transcript-1;
MIAT	myocardial infarction-related transcript;
MRI	magnetic resonance imaging;
NLR	negative likelihood ratio;
PLR	positive likelihood ratio
PRISMA	preferred reporting items for systematic reviews and meta analyses;
PVT1	plasmacytoma variant translocation 1;
qRT-PCR	quantitative reverse transcription polymerase chain reaction;
QUADAS-2	quality assessment of diagnostic accuracy studies 2;
SROC	summary receiver operating characteristic;
TP	true positive;
TN	true negative;
TUG1	taurine-upregulated gene 1.
