# Supplementary material for: Long Non-Coding RNAs as Diagnostic Biomarkers for Ischemic Stroke: A Systematic Review and Meta-Analysis
Source: Genes (Basel). 2024 Dec 18;15(12):1620. doi: 10.3390/genes15121620 (PMC11675862; doi:10.3390/genes15121620)
Supplement: Supplementary file 1 [file genes-15-01620-s001.zip › genes-3290682-supplementary.pdf]

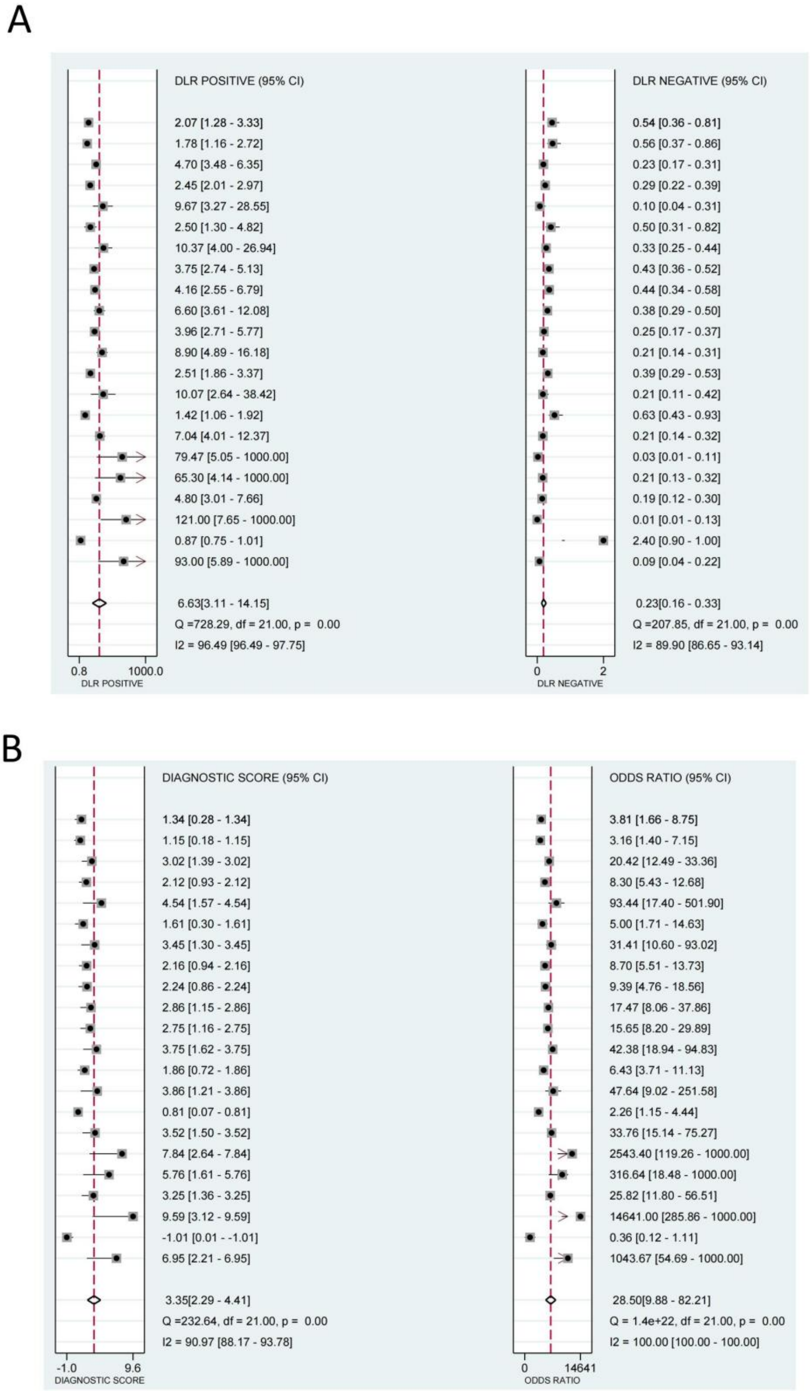

Figure S1. (A) A forest plot for the pooled PLR and NLR estimates of lncRNAs for diagnosing IS. (B) A forest plot for the pooled DOR estimates of lncRNAs for diagnosing IS.

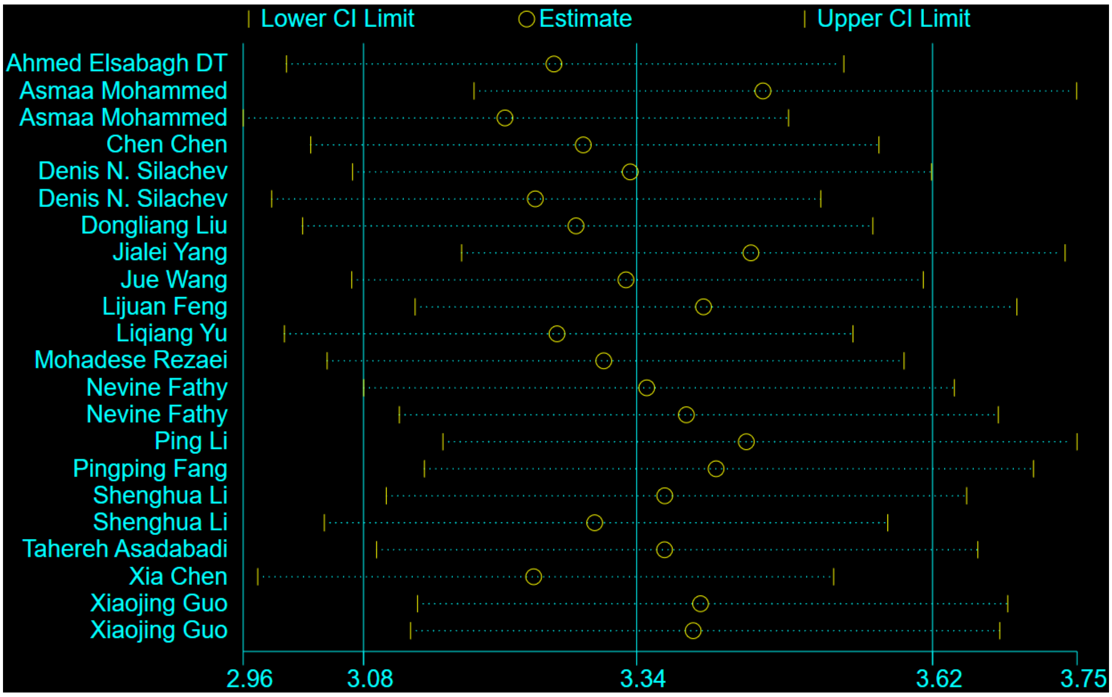

Figure S2. Sensitivity analysis.

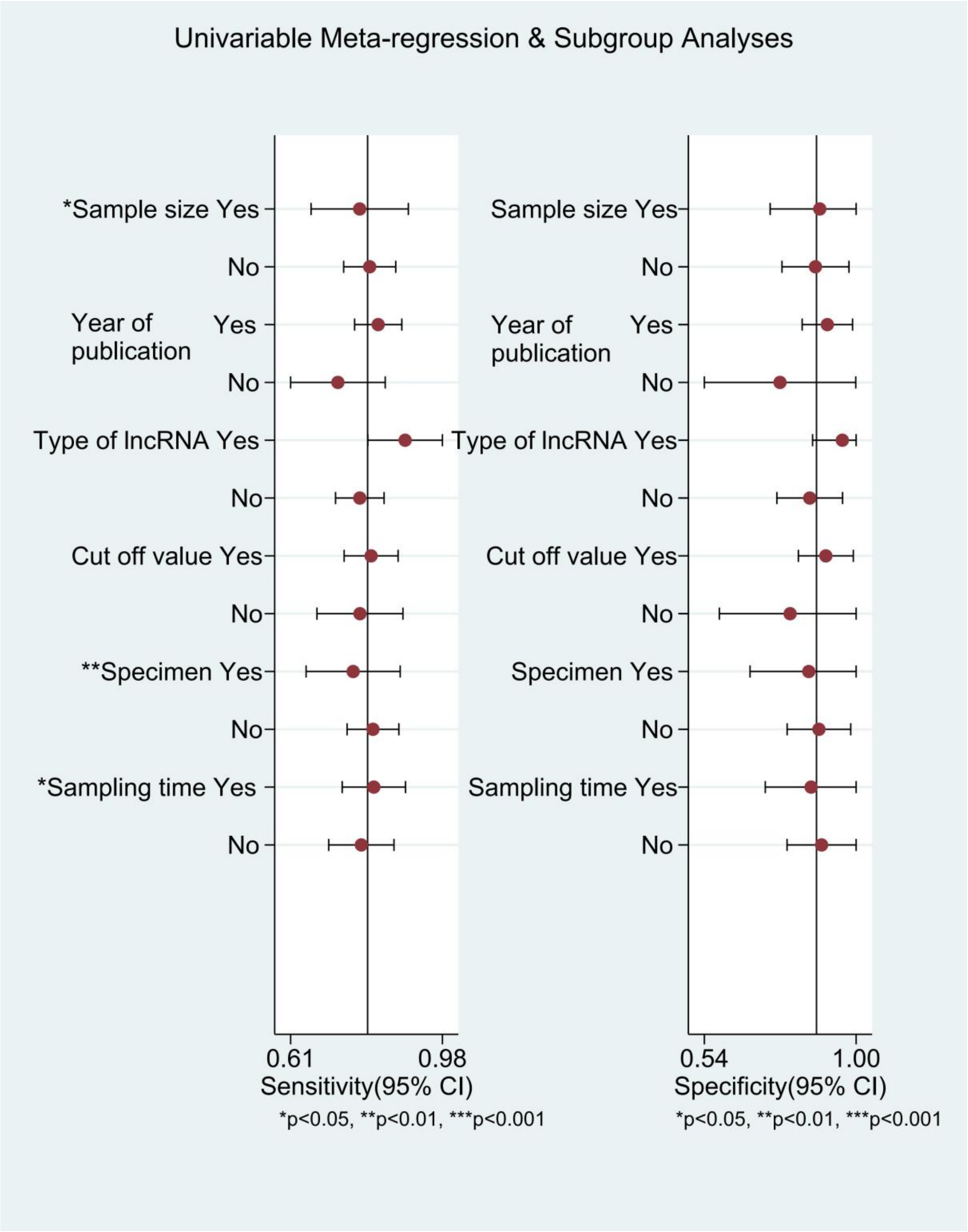

Figure S3. Meta-regression analysis for sensitivity and specificity.
